# Lipoprotein(a) in Disaggregated South Asian Groups: An Analysis of MASALA and the UK Biobank

**DOI:** 10.1016/j.jacadv.2026.103329

**Published:** 2026-10-13

**Authors:** Kavenpreet S. Bal, Mikaila P. Reyes, Alka M. Kanaya, Pam Taub, Michael Y. Tsai, Namratha R. Kandula, Nadia Islam, Michael J. Wilkinson, Mattheus Ramsis, Ehtisham Mahmud, Nilay S. Shah, Anandita Kulkarni, Dinesh Kalra, Harpreet S. Bhatia

**Affiliations:** a Kaiser Permanente Bernard J. Tyson School of Medicine, Pasadena, California, USA; b University of California-San Diego School of Medicine, La Jolla, California, USA; c Department of Medicine, University of California, San Francisco, California, USA; d Division of Cardiovascular Medicine, University of California-San Diego Health, La Jolla, California, USA; e Department of Laboratory Medicine and Pathology, University of Minnesota, Minneapolis, Minnesota, USA; f Department of Preventive Medicine, Northwestern University Feinberg School of Medicine, Chicago, Illinois, USA; g Department of Population Health, NYU Grossman School of Medicine, New York, New York, USA; h Division of Cardiology, Department of Medicine, Northwestern University Feinberg School of Medicine, Chicago, Illinois, USA; i Baylor Scott & White Health, The Heart Hospital, Plano, Texas, USA; j Division of Cardiovascular Medicine, University of Louisville School of Medicine, Kentucky, USA

**Keywords:** lipids, lipoprotein(a), prevention, risk factors, South Asian

## Abstract

**Background:**

South Asian individuals have a high prevalence of elevated lipoprotein(a) [Lp(a)], but little is known about the variation between disaggregated South Asian subgroups.

**Objectives:**

The authors aimed to evaluate Lp(a) levels, nongenetic influences, and the association with incident atherosclerotic cardiovascular disease (ASCVD) in South Asian populations using data from the Meditators of Atherosclerosis in South Asians Living in America (MASALA) and the UK Biobank (UKB) studies.

**Methods:**

Lp(a) levels were assessed by South Asian country of origin and by state among Indian-origin participants. The association between nongenetic factors and Lp(a) levels was evaluated in multivariable linear regression models. Cox proportional hazards models evaluated the association between Lp(a) and ASCVD events in UKB participants.

**Results:**

Among 2,074 MASALA participants born in the South Asian subcontinent (mean age: 56.7 ± 9.6 years), median [Q1-Q3] Lp(a) was 20.7 [10.5-43.0] mg/dL with significant variation by country of origin (*P* = 0.035). Median Lp(a) was 23 [11-48], 19 [10-37], 22 [11-47], and 17 [10-34] mg/dL in participants from Bangladesh, India, Pakistan, and Sri Lanka, respectively. Vegetarian diet, statin use, postmenopausal status, and fewer years in the United States were associated with higher Lp(a) levels while alcohol consumption was associated with lower Lp(a) levels. However, these associations were all very modest. Among 6,845 UKB participants (mean age: 55.1 ± 8.7 years; median Lp(a): 31 [13-65] nmol/L), the associations between Lp(a) and ASCVD did not vary by country of origin or South Asian ethnicity (*P* interaction > 0.05).

**Conclusions:**

There is modest variation in Lp(a) levels, but no difference in the association between Lp(a) and ASCVD outcomes, across South Asian subgroups.

Lipoprotein(a) [Lp(a)] is an independent, causal risk factor for atherosclerotic cardiovascular disease (ASCVD),^1^ with prior studies estimating that 70% to 90% of plasma Lp(a) levels are genetically determined.^2^ Lp(a) levels are observed to vary across race and ethnic groups, possibly related to enrichment in genetic variants that result in higher Lp(a) levels within certain socially-defined groups.^3^ Black and South Asian populations are reported to have the highest levels of Lp(a) among groups with reported Lp(a) levels.^3^ South Asians make up nearly 25% of the world’s population and are one of the fastest growing populations worldwide.^4^ South Asians are at particularly high risk of earlier incidence and higher mortality associated with cardiovascular disease compared with European populations.^4^ Guidelines in the United States note South Asian ancestry as a high-risk ethnicity and an ASCVD-risk-enhancing factor.^5^

However, South Asians are often included in a monolithic Asian group, and there is limited literature on disaggregated South Asian subgroups, including regarding Lp(a) levels. Capturing subgroup-level data enables identification and adjustment for structured heterogeneity that may cluster within subgroups; while Lp(a) is predominantly genetic, evaluating differences by country of origin can help account for within-South Asian diversity and differences that may exist across state and country levels.^6^

We aimed to evaluate variation in Lp(a) levels among disaggregated South Asian subgroups and nongenetic influences on Lp(a) levels in the MASALA (Meditators of Atherosclerosis in South Asians Living in America) study. In addition, we aimed to evaluate the association between Lp(a) levels and ASCVD outcomes among disaggregated South Asian subgroups in the United Kingdom Biobank (UKB).

## Methods

### Study cohorts

The design of the MASALA study has been published previously.^7^ Briefly, the MASALA study is a prospective cohort study of participants of South Asian ancestry of age 40 to 84 years without known ASCVD living in the United States. The initial recruitment occurred from 2010 to 2013, with subsequent recruitments from 2017 to 2018 and 2021 to 2025. Participants were characterized using structured questionnaires, physical examinations, and laboratory and imaging testing. Only participants born in a South Asian country were included in the analysis of Lp(a) levels by South Asian group.

The UKB is a prospective cohort study that enrolled 500,000 individuals across the United Kingdom between the ages of 40 and 69 years between 2006 and 2010. Researchers collected phenotypic and genotypic data about participants from self-completed questionnaires, interviews, physical measurements, and biological samples, with follow-up data extracted from medical records and death certificates. These methods are described in more detail elsewhere.^8^^,^^9^ Two groups of participants with available Lp(a) measures were included from UKB; those born in a South Asian country (India, Pakistan, Bangladesh, Nepal, and Sri Lanka, n = 6,845) and those with self-reported South Asian ethnicity (Indian, Pakistani, and Bangladeshi, n = 6,453). This study was approved by the University of California San Diego Institutional Review Board.

### Lp(a) measurement and outcomes

In MASALA, plasma Lp(a) levels were measured in mass units (mg/dL) using a latex-enhanced turbidometric immunoassay (Denka Seikan).^10^ Furthermore, nongenetic factors, including dietary patterns, alcohol use, tobacco pack year smoking history, statin use, renal disease, liver disease, insulin resistance, menopause, physical activity, and years living in the United States, were evaluated. Dietary pattern was measured using the validated, semiquantitative Food Frequency Questionnaire, which used self-reported dietary habits over the past year to estimate carbohydrate, total fat, saturated fat, monosaturated fat, and polysaturated fat quantitatively in grams (g); allowed participants to self-report a vegetarian diet (yes or no); and calculated percent of total diet from carbohydrates and total fat. Alcohol use was determined by participants’ self-report. Participants reported if they drank alcohol (>1 drink per week) and how much alcohol they consumed, categorized into no consumption, 1 to 7 drinks per week, and >7 drinks per week. Chronic kidney disease stage was categorically identified based on participants’ serum creatinine levels by MASALA investigators. Mean fatty liver attenuation for hepatic steatosis was measured quantitatively via computed tomography-derived imaging of liver fat content through Hounsfield units. Fatty liver was also used as a categorical variable with a Hounsfield unit < 40 identified as fatty liver. Insulin sensitivity was evaluated by fasting serum insulin levels (μIU/mL), 2-hour serum insulin levels (μIU/mL) measured 2 hours after ingestion of 75 g of oral glucose, and insulin sensitivity index, estimated from serum glucose and insulin levels. Postmenopausal status was identified by self-report (yes or no) among female participants. Physical activity was identified by metabolic equivalents of task in minutes per week (continuous). Years in the United States was measured by self-report (continuous). Furthermore, the association between elevated Lp(a) levels of ≥50 mg/dL and family history of myocardial infarction (MI) was evaluated in MASALA using logistic regression with adjustment for age, sex, hypertension, diabetes, total cholesterol, high-density lipoprotein cholesterol (HDL-C), lipid-lowering therapy, and tobacco pack years.

In UKB, plasma Lp(a) levels were measured in molar units (nmol/L) using an immunoturbidimetric assay (Randox).^3^ Lp(a) was log-transformed for the assessment of outcomes. Outcomes evaluated were incident MI, ischemic stroke, and cardiovascular death (CVD), defined as fatal MI or fatal stroke, evaluated individually and as a composite ASCVD outcome. Myocardial infarction was defined as International Classification of Diseases-Tenth Revision codes I20-I25, and ischemic stroke as I63, and the underlying primary cause of death was identified through the Death Registry using International Classification of Diseases-10th Revision codes I20-I25 and I61-I63 to define CVD. Incident events were identified based on data collected during assessment-center visits and national health record linkage.

### Statistical analysis

Baseline characteristics were reported by country of origin in MASALA and UKB. Categorical variables were compared by chi-square tests, and continuous variables were compared using ANOVA or Kruskal-Wallis tests, as appropriate. In MASALA, median Lp(a) levels were assessed by disaggregated groupings, including country, region of India, and state of origin within India. Region of India was divided into North (Chandigarh, Delhi, Haryana, Himachal Pradesh, Punjab, and Rajasthan), Northeast (Arunachal Pradesh, Assam, Manipur, Meghalaya, Mizoram, Nagaland, Sikkim, and Tripura), East (Bihar, Jharkhand, Odisha, and West Bengal), West (Goa, Gujarat, and Maharashtra), Central (Chhattisgarh, Madhya Pradesh, Uttar Pradesh, and Uttarakhand), and South (Andhra Pradesh, Telangana, Karnataka, Kerala, Puducherry, and Tamil Nadu) in accordance with the definitions by the Ministry of Health of the Government of India,^11^ when possible with a sample size ≥10. A sensitivity analysis was performed evaluating Lp(a) levels among nonstatin users with additional stratification by sex.

In MASALA, linear regression models evaluated the association between nongenetic factors and log-transformed Lp(a) levels. Furthermore, the association between elevated Lp(a) levels of ≥50 mg/dL and family history of MI in a first-degree relative was evaluated in MASALA using logistic regression. Models were adjusted for age, sex, hypertension, diabetes, total cholesterol, HDL-C, use of any lipid-lowering therapy, including statins and nonstatins (eg, fibrates), tobacco pack years, estimated glomerular filtration rate, and body mass index.

In UKB, the association between Lp(a) levels (per standard deviation) and incident MI, ischemic stroke, CVD, and composite ASCVD events was evaluated using Cox proportional hazards models. For this analysis, participants with a prior history of ASCVD were excluded. Analyses were stratified by country of birth as well as self-identified ethnicity, which were not mutually exclusive; of those with data for both, there was 96% agreement between country of origin and self-identified ethnicity. Multiplicative interaction between Lp(a) level and South Asian country of birth/ethnicity was also evaluated. Models were adjusted for age, sex, total cholesterol, and HDL-C and for categorical variables including sex, hypertension, diabetes mellitus, current smoking status, use of statins, and body mass index. A sensitivity analysis was performed among nonstatin users. All statistical analyses were performed using R (Version 4.1.2). Statistical significance was defined as a 2-tailed *P* value <0.05.

## Results

Among the 2,208 included MASALA participants, average age was 56.6 ± 9.6 years, 50% (n = 1,105) were female, median Lp(a) was 20 [10-42] mg/dL, and 20% (n = 444) had elevated Lp(a) levels ≥50 mg/dL. Among the 2,077 MASALA participants born on the South Asian subcontinent, 959 (46.3%) were born in India, 524 (25.3%) in Pakistan, 572 (27.6%) in Bangladesh, 18 (0.9%) in Sri Lanka, and 4 (0.2%) from other South Asian countries. Baseline characteristics by South Asian country of origin with ≥10 participants are shown in Table 1. The median Lp(a) level among these MASALA participants was 20 [11-43] mg/dL, and 21% had Lp(a) levels ≥50 mg/dL. The highest levels were among Bangladeshi participants (median: 23 [11-48] mg/dL), followed by Pakistani (22 [11-47] mg/dL), Indian (19 [10-37] mg/dL), and Sri Lankan (17 [10-34] mg/dL], *P* = 0.035 for comparison of all groups) participants (Central Illustration). Lp(a) ≥50 mg/dL was present in 25% of Bangladeshi, 23% of Pakistani, 17% of Indian, and 0% of Sri Lankan participants (*P* < 0.001) (Table 1).

**Table 1:** Baseline Characteristics by South Asian Ethnic Group

	MASALA by Country of Origin						UK Biobank by Country of Origin						
	All (N = 2073)	India (n = 959)	Pakistan (n = 524)	Bangladesh (n = 572)	Sri Lanka (n = 18)	*P* Value	All (N = 6,845)	India (n = 4,158)	Pakistan (n = 1,492)	Bangladesh (n = 259)	Nepal (n = 165)	Sri Lanka (n = 771)	*P* Value
Age, y	56.7 (9.6)	57.0 (9.6)	56.6 (10.0)	56.3 (9.3)	56.9 (7.3)	0.669	55.1 (8.7)	56.7 (8.5)	52.3 (8.2)	50.2 (8.7)	49.1 (7.0)	55.4 (8.4)	<0.001
Female	1,040 (50.2)	452 (47.1)	280 (53.4)	303 (53.0)	5 (27.8)	0.011	3,121 (45.6)	2052 (49.4)	587 (39.3)	80 (30.9)	52 (31.5)	350 (45.4)	<0.001
Lipid-lowering therapy	773 (37.3)	274 (28.6)	192 (36.6)	301 (52.6)	6 (33.3)	<0.001	1,239 (18.1)	711 (17.1)	272 (18.2)	61 (23.6)	11 (6.7)	184 (23.9)	<0.001
Total cholesterol, mg/dL	182 (40)	185 (38)	185 (42)	173 (43)	185 (25)	<0.001	206 (45)	207 (45)	201 (43)	198 (44)	204 (38)	206 (47)	<0.001
HDL cholesterol, mg/dL	49 (13)	50 (14)	49 (12)	47 (11)	49 (12)	0.003	49 (13)	51 (13)	45 (12)	43 (12)	44 (9)	50 (14)	<0.001
LDL cholesterol, mg/dL	107 (34)	110 (32)	110 (34)	100 (35)	113 (23)	<0.001	129 (33)	130 (33)	128 (33)	125 (33)	131 (31)	130 (35)	0.093
Family history of myocardial infarction	855 (41.2)	428 (44.7)	262 (50.0)	155 (27.2)	10 (55.6)	<0.001							
Lp(a), mg/dL	21 [11-43]	19 [10-37]	22 [11-47]	23 [11-48]	17 [10-34]	0.035							
Lp(a) ≥50 mg/dL	425 (21)	161 (17)	123 (23)	141 (25)	0 (0.00)	<0.001							
Lp(a), nmol/L							31 [13-65]	32 [13-66]	29 [13-61]	28 [10-57]	16 [8-43]	39 [15-77]	<0.001
Lp(a) ≥125 nmol/L							477 (9)	306 (9)	89 (7)	15 (7)	10 (8)	57 (9)	0.329
Follow-up time, y							13.33 [12.80-14.11]	13.31 [12.77-14.03]	13.55 [13.04-14.48]	13.46 [12.82-14.13]	13.50 [12.87-14.03]	13.08 [12.66-13.45]	<0.001

Values are n (%), mean (SD), or median [Q1-Q3]

HDL = high-density lipoprotein; LDL = low-density lipoprotein; Lp(a) = lipoprotein(a); MASALA = Mediators of Atherosclerosis in South Asians Living in America; UK = United Kingdom.

**Central Illustration:**
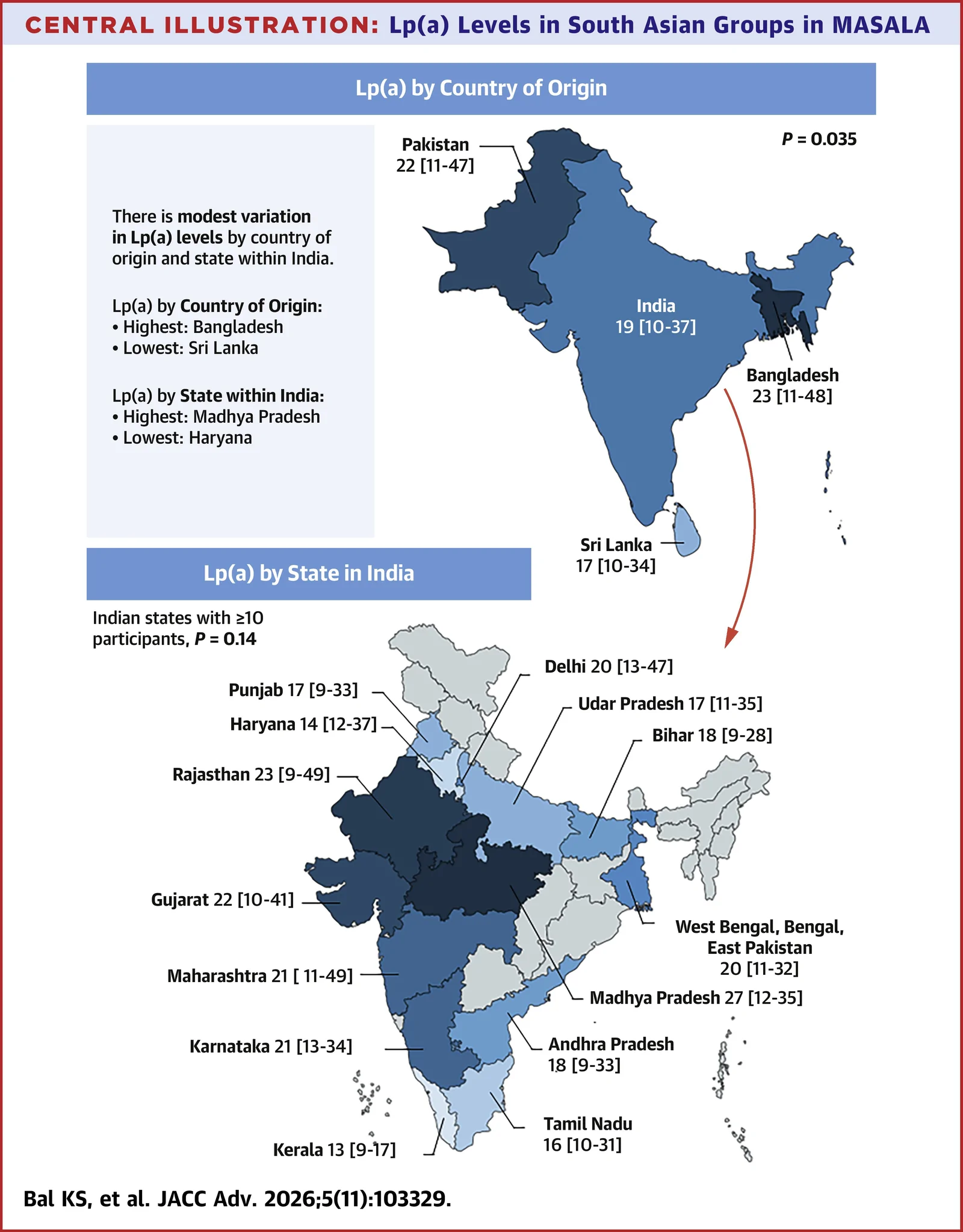
Lp(a) Levels in South Asian Groups in MASALA Lp(a) levels are provided as median [Q1-Q3] in mg/dL by South Asian country of origin and by individual states in India with at least 10 participants from the MASALA cohort. Lighter shading represents lower median Lp(a) levels, whereas darker shading represents higher median Lp(a) levels. Lp(a) = lipoprotein(a); MASALA = Meditators of Atherosclerosis in South Asians Living in America.

Among MASALA participants born in India, there was variation in Lp(a) level by state which did not meet statistical significance (*P* = 0.14). Among states with at least 10 participants, the highest median levels were in those from Madhya Pradesh (n = 21, median: 27 [12-35] mg/dL), and the lowest were observed in those from Kerala (n = 25, median: 13 [9-17] mg/dL) (Central Illustration). There was also variation by region in India which did not meet statistical significance (*P* = 0.07), with the lowest levels among individuals from South India (17 [9-31] mg/dL) and the highest among those from West India (21 [10-44] mg/dL) (Supplemental Table 1).

Among 502,364 UKB participants, 6,845 were born in a South Asian country; 4,158 (60.7%) were born in India, 1,492 (21.8%) in Pakistan, 771 (11.3%) in Sri Lanka, 259 (3.8%) in Bangladesh, and 165 (2.4%) in Nepal. Participant characteristics are shown in Table 1. The median Lp(a) level was 31 [13-65] nmol/L. The highest Lp(a) levels were noted in participants born in Sri Lanka (median: 39 [15-77] nmol/L), followed by India (median: 32 [13-66] nmol/L), Pakistan (median: 29 [13-61] nmol/L), Bangladesh (median: 28 [10-57] nmol/L), and Nepal (median: 17 [8-43] nmol/L). The proportion with Lp(a) > 125 nmol/L was not significantly different by country of birth (*P* = 0.329) (Table 1). A total of 6,453 UKB participants self-identified with South Asian ethnicity: 4,794 (74.3%) were Indian, 1,471 (22.8%) were Pakistani, and 188 (2.9%) were Bangladeshi. The median Lp(a) level was 32 [13-65] nmol/L overall. The highest Lp(a) levels were noted in Indian participants (median: 33 [14-66] nmol/L), followed by Pakistani (29 [13-60] nmol/L) and Bangladeshi participants (29 [11-58] nmol/L) (*P* = 0.02 for comparison). There was no significant difference in the proportion with Lp(a) >125 nmol/L (Supplemental Table 2). Sensitivity analyses excluding participants with baseline statin use demonstrated a similar pattern of Lp(a) levels in both MASALA and UKB, with and without stratification by sex (Supplemental Table 3).

In MASALA, nongenetic associations with Lp(a) levels were evaluated. Higher Lp(a) levels were noted in association with a vegetarian diet (β: 0.10, SE: 0.05, *P* = 0.045), statin use (β: 0.27, SE: 0.13, *P* = 0.04), and postmenopausal status among women (β: 0.26, SE: 0.10, *P* = 0.008). Lower Lp(a) levels were noted with greater alcohol consumption (>1 drink/week vs no consumption: β: −0.16, SE: 0.05, *P* = 0.001; 1-7 drinks/week vs no consumption: β: −0.14, SE: 0.05, *P* = 0.007; >7 drinks/week vs no consumption: β: −0.31, SE: 0.13, *P* = 0.012) and years lived in the United States (β: −0.04, SE: 0.02, *P* = 0.020). No significant associations were observed for other dietary characteristics, tobacco pack year smoking history, renal disease, hepatic steatosis, insulin resistance, or physical activity (Table 2).

**Table 2:** Nongenetic Influences on Lp(a) Levels in MASALA

	n	β	SE	95% CI	*P* Value
Dietary pattern					
Dietary pattern	877	0.04	0.03	−0.02, 0.10	0.17
FFQ energy adjusted carbohydrate (g)	1,112	−0.0005	0.0009	−0.002, 0.001	0.54
FFQ energy adjusted total fat (g)	1,112	0.002	0.002	−0.003, 0.01	0.52
FFQ energy adjusted saturated fat (g)	1,112	0.004	0.006	−0.01, 0.01	0.51
FFQ energy adjusted mono fat (g)	1,112	0.001	0.005	−0.01, 0.01	0.82
FFQ energy adjusted poly fat (g)	1,112	0.01	0.01	−0.01, 0.02	0.50
Vegetarian diet (yes/no)	**877**	**0.10**	**0.05**	**0.002, 0.20**	**0.045**
Dietary Quality AHEI-2010 Component Score	877	−0.0004	0.004	−0.01, 0.01	0.92
% Carbohydrates (% of total KCal)	1,112	−0.001	0.004	−0.01, 0.01	0.82
% Total fat (% of total KCal)	1,112	0.005	0.004	−0.004, 0.01	0.28
Alcohol use					
Consumes >1 drink per week (yes)	2,196	**−0.16**	**0.05**	**−0.26, −0.06**	**0.001**
Alcohol consumption	2,196				
No consumption		REF	--	--	--
1-7 Drinks per week		**−0.14**	**0.05**	**−0.24, −0.04**	**0.007**
>7 Drinks per week		**−0.31**	**0.13**	**−0.56, −0.07**	**0.012**
Smoking					
Tobacco pack years	2,196	−0.002	0.003	−0.007, 0.004	0.55
Medication use					
Statin use	2,196	**0.27**	**0.13**	**0.01, 0.53**	**0.04**
Renal disease					
CKD stage (categorical)	2,196	−0.01	0.05	−0.10, 0.08	0.86
Hepatic steatosis					
Mean liver fat attenuation (HU)	1,124	0.0003	0.002	−0.004, 0.005	0.90
Fatty liver (HU <40; yes/no)	1,124	−0.09	0.07	−0.23, 0.05	0.20
Insulin resistance					
Insulin sensitivity index	842	0.001	0.01	−0.01, 0.01	0.87
Fasting serum insulin (μIU/mL)	841	−0.0002	0.0003	−0.0003, 0.001	0.40
2-h Serum insulin (μIU/mL)	670	0.00003	0.00005	−0.0001, 0.0001	0.59
Menopause					
Postmenopausal (yes)	**543**	**0.26**	**0.10**	**0.07, 0.46**	**0.008**
Physical activity					
Physical activity (MET min/wk)	2,196	−0.000004	0.000004	−0.00001, 0.000003	0.27
Exercise (MET min/wk)	2,196	0.00001	0.00001	−0.00002, 0.00003	0.72
Years in the United States	**2,115**	**−0.04**	**0.02**	**−0.07, −0.006**	**0.020**

Multivariable models were adjusted for age, gender, hypertension, diabetes, total cholesterol, HDL-C, lipid-lowering therapy, tobacco pack years, eGFR, and BMI. **Bold** values are for significant values with *P* < 0.05, but bolding can be removed

BMI = body mass index; CKD = chronic kidney disease; eGFR = estimated glomerular filtration rate; FFQ = Food Frequency Questionnaire; HU = Hounsfield units; METs = metabolic equivalents of tasks; other abbreviations as in Table 1.

In MASALA, among 1,762 participants with an Lp(a) <50 mg/dL, 737 (41.8%) reported a family history of MI, whereas among 443 participants with an Lp(a) >50 mg/dL, 193 (43.6%) reported a family history of MI (*P* = 0.51). No significant association was found between Lp(a) level and family history of MI after adjusting for covariates (OR: 1.00; 95% CI: 0.81-1.24; *P* = 0.97).

In the UKB, 260 (3.8%) participants had an incident MI, 83 (1.2%) an ischemic stroke, 71 (1.0%) had a CVD, and 356 (5.2%) any ASCVD event. Among all participants with a South Asian country of origin, higher Lp(a) was associated with increased risk of MI (HR per SD: 1.14; 95% CI: 0.99-1.31), CVD (HR per SD: 1.20; 95% CI: 0.93-1.53), and composite ASCVD (HR per SD: 1.09; 95% CI: 0.97-1.22) with borderline statistical significance, but not ischemic stroke (HR per SD: 1.00; 95% CI: 0.81-1.25) (Figure 1) in multivariable adjusted models. No significant multiplicative interaction between Lp(a) level and South Asian country of origin was noted for any outcome. Among participants of self-identified South Asian ethnicity in UKB, Lp(a) was significantly associated with MI (HR per SD: 1.25; 95% CI: 1.10-1.43) and composite ASCVD (HR per SD: 1.13; 95% CI: 1.01-1.27), but not ischemic stroke (HR per SD: 0.94; 95% CI: 0.75-1.19) or CVD (HR per SD: 1.06; 95% CI: 0.82-1.37) (Supplemental Figure 1) in multivariable adjusted models. No significant multiplicative interaction between Lp(a) level and South Asian ethnicity was noted for any outcome.

**Figure 1:**
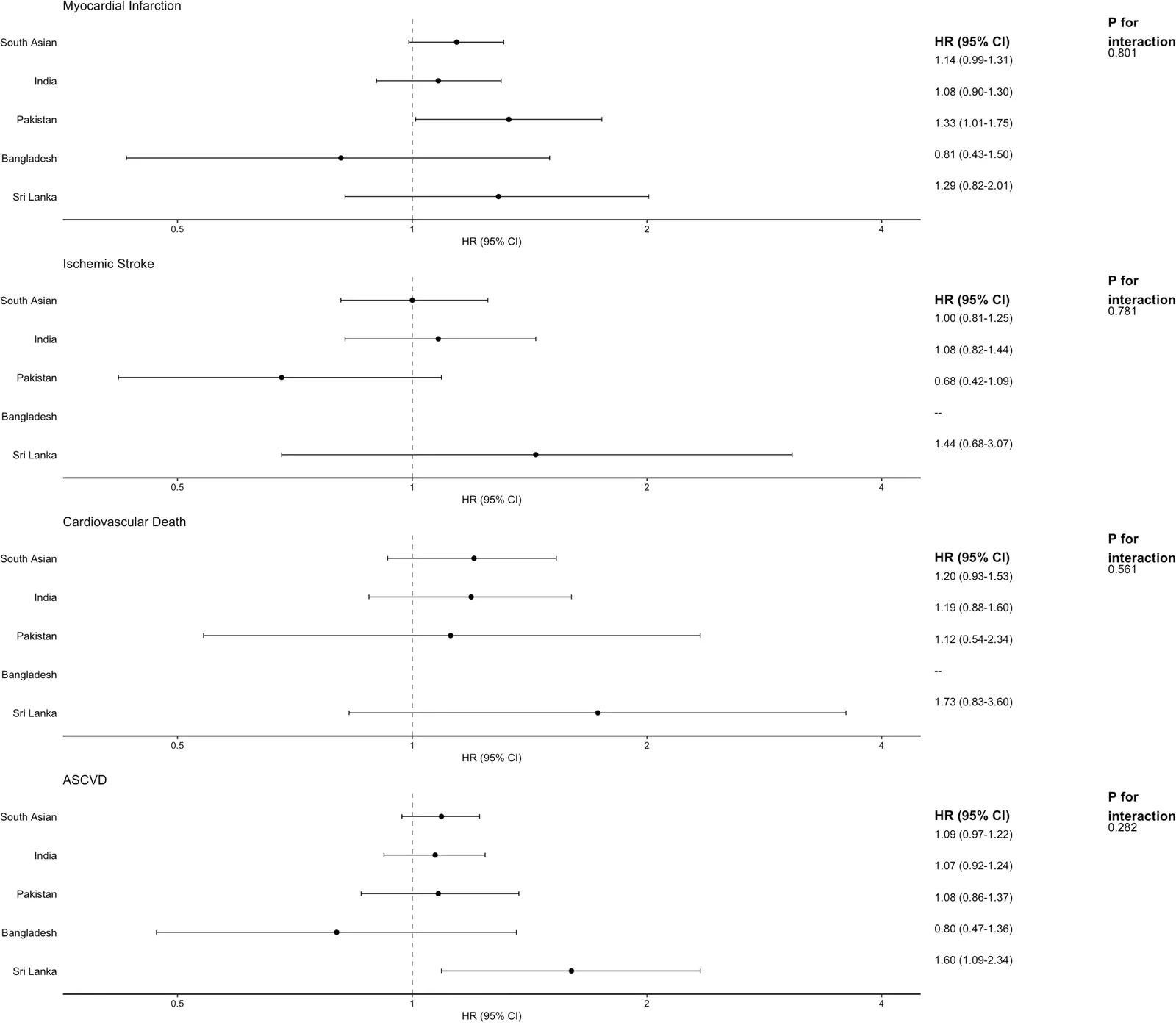
Lipoprotein(a) and Cardiovascular Outcomes in South Asian Groups in UKB Adjusted hazard ratios are presented per standard deviation of lipoprotein(a) from the UKB cohort by country of origin. Models are adjusted for age, sex, total cholesterol, high-density lipoprotein cholesterol, hypertension, diabetes mellitus, current smoking, cholesterol medication, and body mass index. Participants from Nepal were not included because of inadequate sample sizes; participants from Bangladesh were not included for ischemic stroke and cardiovascular death because of insufficient sample sizes. ASCVD = atherosclerotic cardiovascular disease; UKB = United Kingdom Biobank.

## Discussion

This study evaluated Lp(a) levels in disaggregated South Asian origin subgroups in 2 large cohorts in the United States (MASALA) and the United Kingdom (UKB). A modest variation in Lp(a) levels was identified by country, region, and Indian state in South Asia, but the association between Lp(a) and cardiovascular events did not vary by disaggregated South Asian subgroups in UKB. Some nongenetic factors, including vegetarian diet, statin use, postmenopausal status, and fewer years living in the United States, were associated with higher Lp(a) levels among MASALA participants while alcohol use was associated with lower Lp(a) levels. These findings emphasize the importance of a further study of disaggregated South Asian subgroups rather than treating South Asia or Asia as a singular population.

In MASALA, a modest variation in Lp(a) levels was noted by region of India, varying from median 17 mg/dL in the South to 21 mg/dL in the West. A more pronounced variation was noted by state from 14 mg/dL in Haryana to 27 mg/dL in Madhya Pradesh. Most participants in MASALA were from the West (43.5%) and South (23.4%) regions, illustrating the need for further evaluation with larger sample sizes from states in the Northeast, East, Central, and Northern regions.

While 70% to 90% of plasma Lp(a) levels are estimated to be genetically determined, we observed modest nongenetic influences on Lp(a) levels in South Asian individuals. While other studies suggest a vegetarian diet does not affect Lp(a) levels or may even have a favorable impact,^12^ our study suggests South Asians with a vegetarian diet have modestly higher Lp(a) levels.^13^ The reasons for this are unclear and require further exploration; however, prior studies suggest that less saturated-fat intake and higher carbohydrate intake, which may be seen in vegetarian diets,^14^ are associated with higher Lp(a) levels.^15^^,^^16^ Our findings regarding alcohol consumption are consistent with those of earlier studies demonstrating that those who did not consume alcohol had higher median Lp(a) levels than the lowest, middle, and highest alcohol consumers.^17^ Further investigation into the mechanism of this association is warranted. Our finding that statin use was associated with a higher Lp(a) level was consistent with prior studies showing that statins are associated with a modest, but typically clinically insignificant, increase in Lp(a).^5^^,^^18^ Postmenopausal status was associated with higher Lp(a) levels, as described in other populations.^19^, ^20^, ^21^ More years living in the United States was associated with lower Lp(a) levels, which is likely multifactorial, including differences in immigration waves to the United States and possibly other unmeasured confounding factors. Importantly, all associations between nongenetic influences and Lp(a) levels were very modest, whereas metabolic factors (eg, hepatic steatosis and renal disease) were not associated with elevated Lp(a) levels, consistent with prior literature.^1^ The lack of significant association between Lp(a) and family history of MI in MASALA was likely multifactorial: First, there was limited sample size of individuals with elevated Lp(a) levels and a family history of MI; second, MI in a first-degree family member is a self-reported measure and may underestimate true risk; third, participants did not specifically indicate whether they had a family history of premature MI, which may be more closely related to genetic factors such as Lp(a).

Lp(a) is a well-established risk factor for MI^22^ and coronary artery disease.^23^ When evaluated by country of origin in UKB, Lp(a) was borderline significantly associated with MI and composite ASCVD; when evaluated by ethnicity, Lp(a) was significantly associated with both MI and composite ASCVD. There was no significant multiplicative interaction between Lp(a) and country of origin or ethnicity for any outcome evaluated. Thus, while Lp(a) levels vary by South Asian country of origin and ethnicity, the association between Lp(a) and cardiovascular disease risk did not appear to differ.

We observed a different pattern of distribution of Lp(a) levels by country of origin between MASALA and UKB. In MASALA, the highest levels were among those from Bangladesh, while in UKB, the highest levels were among those from Sri Lanka. Differences between individuals from India, Pakistan, and Bangladesh were small in both studies. There are several potential explanations for the difference among participants from Sri Lanka. These findings are likely due to the small number of participants of Sri Lankan origin in MASALA and limited power compared to UKB. In addition, this may illustrate the limitations of using country of origin to understand the variation in Lp(a) levels. There may also be differences between South Asian populations in the United Kingdom and the United States because of distinct migration patterns, which may contribute to heterogeneity. Measured or unmeasured nongenetic components may be driving some of these differences. In addition, differences in the proportion of men and women from each country likely drive some of these differences as women generally have higher Lp(a) levels than men. For example, among participants not on baseline lipid-lowering therapy, 66% of male participants were from India in UKB compared with 51% of MASALA, and 3% were from Bangladesh in UKB while 21% were from Bangladesh in MASALA. Despite these differences, the overall country-level analyses generally demonstrate modest differences in median Lp(a) levels, with the exception of individuals born in Sri Lanka and Nepal. This remained consistent in a sensitivity analysis excluding participants on baseline lipid-lowering therapy.

### Study limitations

Our study has notable limitations. While we evaluated the distribution of Lp(a) levels in 2 separate cohorts of South Asians in the United Kingdom and United States, both studies utilized different forms of measuring Lp(a), which cannot reliably be converted to one another.^24^ In both cohorts, country of origin was used for the primary analysis, which may not fully capture ethnic diversity. However, in UKB, results were consistent when using self-reported ethnicity instead. Despite large overall sample sizes, the majority of participants were from India, and certain individual groups, such as individuals from Sri Lanka and other South Asian countries, had relatively small sample sizes. We may also have had limited power to examine whether the association between Lp(a) and ASCVD events varied by South Asian country in the UKB. In addition, the study populations were not enriched with individuals with elevated Lp(a) levels, which may also have limited our ability to detect differences in ASCVD outcomes by country. While sample size is a limitation of the present analysis, this highlights the need for continued research in this area. In particular, there is a need for large, prospective or cross-sectional analyses across diverse groups within South Asia for further understanding of variation in risk among more specific South Asian groups, especially given the high risk of premature ASCVD in this population. Lastly, our study evaluates Lp(a) levels primarily among participants who have immigrated from their country of origin; studies evaluating broader groups of South Asian individuals, including with varying levels of education, income, and diversity of lifestyle factors, are needed.

## Conclusions

While Lp(a) levels are on average higher in South Asians than in other ethnic groups, our study identifies a modest variation in Lp(a) levels within South Asia. However, the association between Lp(a) and cardiovascular outcomes did not vary by country of origin. These findings add to existing literature about Lp(a) levels and cardiovascular risk in South Asian groups and emphasize the need for a future study with larger sample sizes to further evaluate differences in Lp(a) levels by disaggregated South Asian subgroups and a greater variety of cardiovascular outcomes.

## Funding support and author disclosures

This study was funded by UC San Diego BEACON. Dr Bhatia was supported by 10.13039/100000002National Institutes of Health, Grant 1K08HL166962. Dr Bhatia has reported consulting/advisory relationships with Abbott, Arrowhead, Bayer, Kaneka, Merck, Novartis, and NewAmsterdam. The content is solely the responsibility of the authors and does not necessarily represent the official views of the National Heart, Lung, and Blood Institute or the National Institutes of Health. All other authors have reported that they have no relationships relevant to the contents of this paper to disclose.
