# Supplementary material for: Lipoprotein(a) in Disaggregated South Asian Groups: An Analysis of MASALA and the UK Biobank
Source: JACC Adv. 2026 Oct 13;5(11):103329. doi: 10.1016/j.jacadv.2026.103329 (PMC13685961; doi:10.1016/j.jacadv.2026.103329)
Supplement: Supplementary file 1 [file mmc1.docx]

**Supplemental Table 1:** Lipoprotein(a) Levels by Region, Country, and State in MASALA

| **Group** | **Sample Size** | **Lp(a) Level,**  median [IQR], mg/dL | **p-value** |
| --- | --- | --- | --- |
| **South Asian Country** |  |  | 0.04 |
| India | 959 | 19 [10-37] |  |
| Bangladesh | 572 | 23 [11-48] |  |
| Pakistan | 524 | 22 [11-47] |  |
| Sri Lanka | 18 | 17 [10-34] |  |
| Other South Asian country | 4 | 9 [9-23] |  |
| **Region of India** |  |  | 0.07 |
| North | 165 | 18 [9-35] |  |
| East | 59 | 18 [9-31] |  |
| West | 412 | 21 [10-44] |  |
| Central | 86 | 18 [11-35] |  |
| South | 222 | 17 [9-31] |  |
| **State in India** |  |  | 0.17 |
| Gujarat | 267 | 22 [10-41] |  |
| Maharashtra | 144 | 21 [11-49] |  |
| Punjab | 89 | 17 [9-33] |  |
| Tamil Nadu | 79 | 16 [10-31] |  |
| Andra Pradesh | 77 | 18 [9-33] |  |
| Udar Pradesh | 64 | 17 [11-35] |  |
| Delhi | 49 | 20 [13-47] |  |
| Karnataka | 40 | 21 [13-34] |  |
| Bengal, West Bengal, East Pakistan | 40 | 20 [11-32] |  |
| Kerala | 25 | 13 [9-17] |  |
| Madhya Pradesh | 21 | 27 [12-35] |  |
| Bihar | 15 | 18 [9-28] |  |
| Rajasthan | 12 | 23 [9-49] |  |
| Haryana | 10 | 14 [12-37] |  |
| Other Indian State | 27 | 23 [11-34] |  |

Abbreviations: Lp(a), Lipoprotein(a)

For results by state in India, statistical significance was tested among states with at least 10 participants. States with <10 participants were combined into “Other Indian State”.

**Supplemental Table 2:** Baseline Characteristics by South Asian Ethnicity in UK Biobank

|  | UK Biobank by Ethnicity | | | | |
| --- | --- | --- | --- | --- | --- |
|  | All  n=6453 | Indian  n=4794 | Pakistani n=1471 | Bangladeshi n=188 | P |
| Age, years | 53.3 (8.4) | 54.1 (8.3) | 51.3 (8.3) | 49.7 (8.8) | <0.001 |
| Female | 2972 (46.1) | 2339 (48.8) | 574 (39.0) | 59 (31.4) | <0.001 |
| Lipid lowering therapy | 1084 (16.8) | 772 (16.1) | 265 (18.0) | 47 (25.0) | 0.002 |
| Total cholesterol, mg/dL | 205 (43) | 206 (43) | 201 (43) | 199 (44) | <0.001 |
| HDL-C mg/dL | 49 (12) | 50 (12) | 45 (11) | 44 (12) | <0.001 |
| LDL-C, mg/dL | 129 (32) | 129 (32) | 128 (32) | 126 (33) | 0.08 |
| Lp(a), nmol/L | 32 [13, 65] | 33 [14, 66] | 29 [13, 60] | 29 [11, 58] | 0.02 |
| Lp(a) >125 nmol/L | 505 (7.8) | 389 (8.1) | 102 (6.9) | 14 (7.4) | 0.33 |
| Follow-up time, years | 13.21 [12.75, 13.79] | 13.54 [13.02, 14.50] | 13.38 [12.81, 14.14] | 13.21 [12.75, 13.79] | <0.001 |

Abbreviations: HDL-C, high-density lipoprotein cholesterol; Lp(a), Lipoprotein (a); LDL-C, low-density lipoprotein cholesterol

**Supplemental Table 3:** Lp(a) Levels in Participants in MASALA and UK Biobank without Baseline Lipid Lowering Therapy by Country of Origin

|  | MASALA | | UK Biobank | |
| --- | --- | --- | --- | --- |
|  | n (%) | Lp(a), mg/dL | n (%) | Lp(a), nmol/L |
| **By country of origin** |  |  |  |  |
| All | 1300 (100%) | 20 [10, 40] | 4499 (100%) | 31 [13, 65] |
| India | 685 (53%) | 19 [9, 36] | 2756 (61%) | 32 [13, 66] |
| Pakistan | 332 (26%) | 22 [10, 42] | 983 (22%) | 29 [13, 61] |
| Bangladesh | 271 (21%) | 23 [11, 50] | 167 (4%) | 24 [10, 53] |
| Sri Lanka | 12 (1%) | 21 [14, 43] | 469 (10%) | 38 [15, 75] |
| Nepal | -- | -- | 124 (3%) | 16 [8, 44] |
| p |  | 0.275 |  | <0.001 |
|  |  |  |  |  |
| **By sex** |  |  |  |  |
| **Female** |  |  |  |  |
| All | 671 (100%) | 21 [11, 41] | 2470 (100%) | 34 [14, 70] |
| India | 343 (51%) | 20 [12, 38] | 1620 (66%) | 34 [14, 69] |
| Pakistan | 185 (28%) | 21 [10, 42] | 469 (19%) | 31 [15, 66] |
| Bangladesh | 143 (21%) | 23 [11, 55] | 69 (3%) | 23 [13, 59] |
| Sri Lanka | -- | -- | 272 (11%) | 42 [16, 86] |
| Nepal | -- | -- | 40 (2%) | 20 [9, 38] |
| p |  | 0.293 |  | 0.001 |
|  |  |  |  |  |
| **Male** |  |  |  |  |
| All | 617 (100%) | 20 [9, 39] | 2029 (100%) | 27 [12, 58] |
| India | 342 (55%) | 18 [9, 33] | 1136 (46%) | 27 [12, 58] |
| Pakistan | 147 (24%) | 22 [11, 41] | 514 (25%) | 27 [12, 59] |
| Bangladesh | 128 (21%) | 22 [10, 45] | 98 (5%) | 27 [10, 52] |
| Sri Lanka | -- | -- | 197 (10%) | 31 [14, 59] |
| Nepal | -- | -- | 84 (4%) | 14 [8, 56] |
| P-value |  | 0.189 |  | 0.032 |

**Supplemental Figure 1.** Lp(a) and Cardiovascular Outcomes in UKB by South Asian Ethnicity

Hazard Ratios are presented per standard deviation of Lp(a). Models are adjusted for age, sex, total cholesterol, high-density lipoprotein cholesterol, hypertension, diabetes mellitus, current smoking, cholesterol medication, and body mass index. Participants from Nepal were not included due to inadequate sample sizes; participants from Bangladesh were not included for ischemic stroke and cardiovascular death due to insufficient sample sizes.
